# Protocol for nuclear dissociation of the adult *C. elegans* for single-nucleus RNA sequencing and its application for mapping environmental responses

**DOI:** 10.1016/j.xpro.2023.102756

**Published:** 2023-12-02

**Authors:** Max T. Levenson, Rio Barrere-Cain, Lisa Truong, Yen-Wei Chen, Karissa Shuck, Blake Panter, Ella Reich, Xia Yang, Patrick Allard

**Affiliations:** 1 Molecular Toxicology Inter-Departmental Program, UCLA, Los Angeles, CA 90095, USA; 2 Institute for Society & Genetics, UCLA, Los Angeles, CA 90095, USA; 3 Human Genetics Graduate Program, UCLA, Los Angeles, CA 90095, USA; 4 Integrative Biology and Physiology Department, UCLA, Los Angeles, CA 90095, USA; 5 Molecular Biology Institute, UCLA, Los Angeles, CA 90095, USA

**Keywords:** Single Cell, RNA-seq, Model Organisms, Gene Expression

## Abstract

*Caenorhabditis elegans* is a valuable model to study organ, tissue, and cell-type responses to external cues. However, the nematode comprises multiple syncytial tissues with spatial coordinates corresponding to distinct nuclear transcriptomes. Here, we present a single-nucleus RNA sequencing (snRNA-seq) protocol that aims to overcome difficulties encountered with single-cell RNA sequencing in *C. elegans*. We describe steps for isolating *C. elegans* nuclei for downstream applications including snRNA-seq applied to the context of alcohol exposure.

For complete details on the use and execution of this protocol, please refer to Truong et al. (2023).^1^

## Highlights

• Whole-body nuclear dissociation of *C. elegans* for single-nucleus RNA sequencing
• Detailed steps for *C. elegans* alcohol liquid exposure for snRNA-seq applications
• Worm growth conditions to obtain the F1 and F3 generations after exposure in the P0

## Abstract (editor highlights)

Publisher’s note: Undertaking any experimental protocol requires adherence to local institutional guidelines for laboratory safety and ethics.

## Graphical abstract

**Figure:**
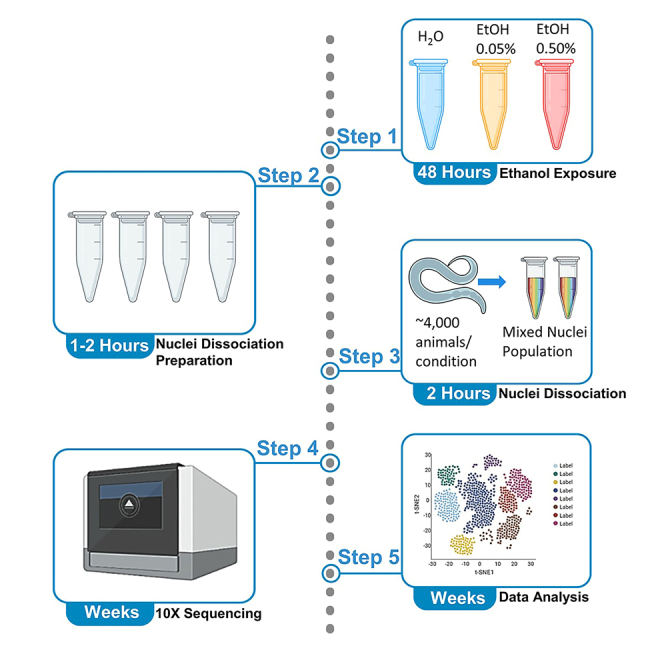

## Before you begin

The identification of cell-specific responses to environmental factors has long been unattainable using conventional bulk RNA-sequencing. By contrast, single-cell RNA sequencing has enabled a deeper examination of tissue and cellular heterogeneity under normal conditions or under stress, including environmental stress.^2^^,^^3^ Single cell approaches have been applied to the model organism *Caenorhabditis elegans* (*C. elegans*) to comprehensively capture the transcriptome of individual cells but at the scale of a whole organism.^4^^,^^5^^,^^6^ However, up to 30% of all somatic cells in the nematode are multinucleated while the adult germline is also primarily syncytial.^7^^,^^8^ Thus, single cell techniques are not able to capture the complexity of such organs, tissues, and cell types or their response to environmental cues. We recently published the development and application of a single nucleus dissociation followed by single nucleus RNA sequencing (snRNA-seq) at the scale of the entire adult *C. elegans*.^1^ This approach was not only used to capture the transcriptome of all major cell types in the nematode but also to identify the heterogeneity in cell-specific responses to ethanol across generations. The protocol presented here covers the key aspects of the methodology, including the exposure protocol that was followed as well as the isolation of high-quality nuclei from the adult *C. elegans*.

### Animal synchronization

**Timing: 1 week**

1. Set up 10–15 60 mm OP50 seeded NGM plates (see the note below) with 5–6 L4 animals per plate.
2. After 5 days at 20°C, when the next generation of adults with embryos are present but **not starved**, proceed to “rapid bleach to synchronize animals for exposure”.

***Note:*** For OP50 growth conditions and seeding protocol see Stiernagle, 2006^9^**.** Ensure plates will not starve due to high plating density.

***Note:*** Animal plating densities may vary based on what strain is being grown and the amount of OP50 seeded on each plate.

***Note:*** Ensure ∼4,000 animals per condition can be harvested.

### OP50 + M9 preparation

**Timing: 24 h**

3. Pick a single colony of OP50 bacteria into a sterile 1 L Erlenmeyer flask containing 100 mL Luria Broth and culture overnight shaking at 37°C.
4. Transfer the culture evenly between 3 sterile and pre-weighed 50 mL tubes.
5. Spin down the culture at 4000 × *g* for 5 min and remove the supernatant.
6. Re-weigh each tube and calculate the weight of the bacterial pellet.
7. Dilute the pellet with M9 (store at room temperature, up to a year) to a stock concentration of 100 mg/mL OP50 + M9.

### Nuclei dissociation reagent preparation

**Timing: 1 h**

8. The day of “nuclei dissociation”.
  a. Label all necessary tubes.
  b. Dilute DAPI (or Hoechst) stain (stock concentration of 2.5 mg/mL DAPI, store at 4°C up to a month).
  c. Make Modified FA buffer (modified from Jonge et al.*,* 2020^10^). Buffer can be stored at 4°C for up to 2 weeks; however, you must add protease and RNase inhibitors fresh.
  d. Make RNase free 1× PBS by diluting 10× PBS with commercially available DEPC-treated water (see “key resources table”) to 1× PBS, ensuring a volume of 2 mL per condition.
  e. Make 1 mL of RNase free water containing protease inhibitor tablet(s) (according to manufacturer’s instructions). Keep on ice, use the same day.

## Key resources table

**Table:** 

REAGENT or RESOURCE	SOURCE	IDENTIFIER
**Chemicals, peptides, and recombinant proteins**		

RNase-free water, DEPC-treated	Fisher Scientific	# BP2484100
RNase Zap	Fisher Scientific	# AM9780
RNase inhibitor	Thermo Fisher Scientific	# 10777019
Protease inhibitor	Roche	# 11697498001
Bovine serum albumin (BSA)	Fisher Scientific	# BP1600-100
PBS 10× RNase-free pH 7.4	Thermo Fisher Scientific	# AM9624
HEPES 1 M, pH 7.5	Thermo Fisher Scientific	#J60712.AK
EDTA 0.5 M, pH 8.0	Thermo Fisher Scientific	# AM9260G
Triton X-100	Fisher Scientific	# BP151-100
NaCl 5 M	Fisher Scientific	# AM9759
200 Proof ethanol, molecular biology grade	Fisher Scientific	# 64-17-5
DAPI	Sigma-Aldrich	# 10236276001
Sodium hydroxide (NaOH)	Fisher Scientific	# BP359-500
Hypochlorite (Essendant Clorox germicidal bleach)	Fisher Scientific	# 50371500

**Deposited data**		

Raw snRNA-seq repository	This paper	https://singlecell.broadinstitute.org/single_cell/study/SCP922/single-nucleus-resolution-mapping-of-the-adult-c-elegans-and-its-application-to-elucidate-inter-and-trans-generational-response-to-alcohol
Raw snRNA-seq repository (alternate location)	This paper	https://www.ncbi.nlm.nih.gov/geo/query/acc.cgi?acc=GSE208229

**Experimental models: Organisms/strains**		

*C. elegans*: JK560 [*fog-1(q253)*I]	Caenorhabditis Genetics Center (CGC)	JK560

**Software and algorithms**		

Debris Identification using Expectant Maximization (DIEM)	Alvarez et al.^11^	https://github.com/marcalva/diem
SoupX	Young et al.^12^	https://github.com/constantAmateur/SoupX
Cell Ranger Pipeline	10× Genomics	https://support.10xgenomics.com/single-cell-gene-expression/software/pipelines/latest/installation

**Other**		

1.5 mL low-retention tubes (RNase-free)	Thermo Fisher Scientific	# 3448
10.0 μM pore size, hydrophilic nylon filters	EMD Millipore	# NY1002500
Swinnex filter holder, 25 mm	EMD Millipore	# SX00025000
15 mL RNase-free tubes	VWR	# 76176-950
50 mL RNase-free tubes	VWR	# 76176-952
10 μL pipette tips, low bind	Genesee Scientific	# 23-201
200 μL pipette tips, low bind	Genesee Scientific	# 23-412
1,000 μL pipette tips, low bind	Genesee Scientific	# 23-430
Flowmi 40 μm filters	Sigma-Aldrich	# BAH136800040-50EA
DWK Life Sciences Wheaton Dounce tissue grinder	Fisher Scientific	# 06-434
Fisherbrand pellet pestle	Fisher Scientific	# 12-141-363
10 mL plastic pipette	Bioland	# 327001
96 well flat-bottom plates	Genesee Scientific	# 25-104

## Materials and equipment

**Table:** Modified FA Buffer

Reagent	Final concentration	Amount
HEPES pH 7.5 (1 M)	50 mM	200 μL
EDTA (0.5 M)	1 mM	8 μL
Triton X-100 (10%)	0.1%	40 μL
NaCl (5 M)	150 mM	120 μL
Protease Inhibitor (50×)	0.5×	40 μL
RNase Inhibitor (40 U/μL)	0.2 U/μL	20 μL
RNase Free Water		to 4 mL
**Total**		**4 mL**

Store at 4°C for up to 2 weeks.

**Table:** Bleach Solution

Reagent	Amount
Hypochlorite 8.25% (Clorox Concentrated)	3.9 mL
10 N NaOH	7.5 mL
dH_2_O	38.6 mL
**Total**	**50 mL**

**CRITICAL:** Prepare bleach solution fresh, it can be prepared in smaller batches, keep at room temperature. Hypochlorite and Sodium Hydroxide are corrosives that can damage skin and eyes if they come into contact. Handle with caution utilizing proper PPE including a chemical resistant lab coat, goggles, and gloves.

## Step-by-step method details

### Rapid bleach to synchronize animals for exposure

**Timing: 1 h**

**Figure 1:**
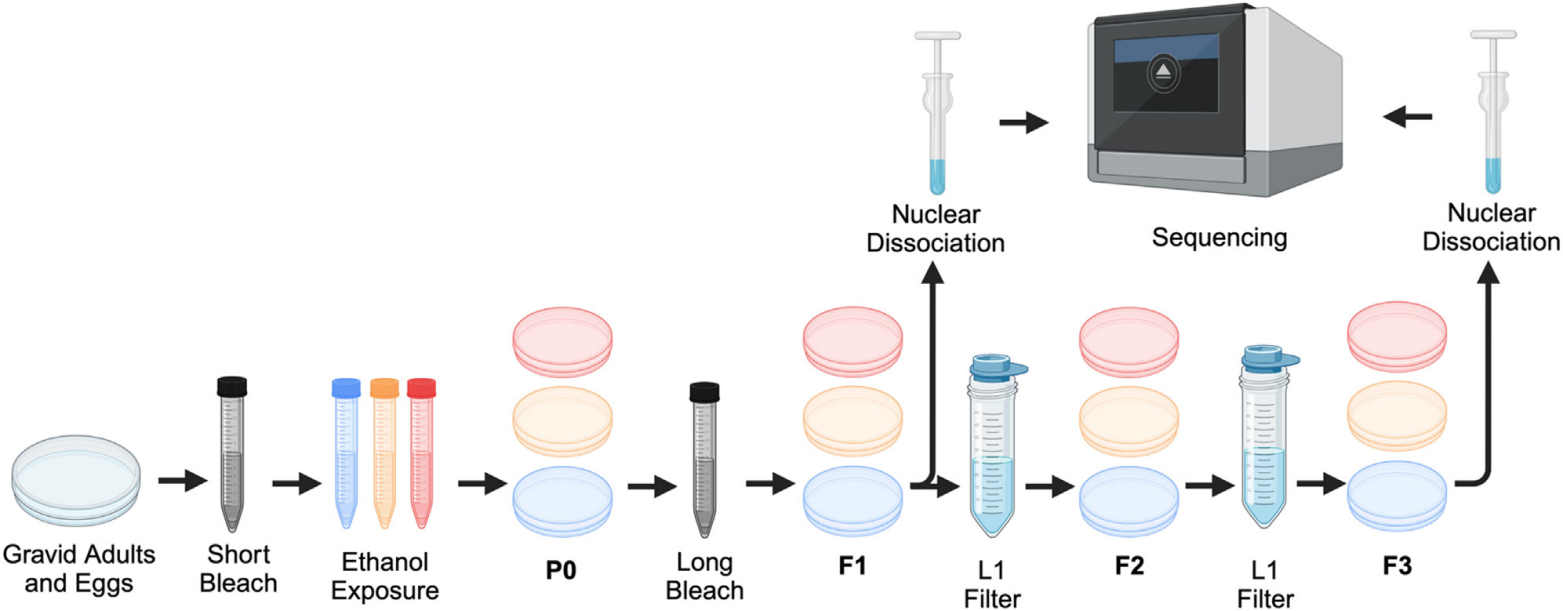
Flowchart overview of exposure, maintenance, and nuclear dissociation

This step will synchronize animals in preparation for ethanol exposure. At this stage, the animals should be gravid adults and the plates should also contain hundreds of eggs (Figure 1).

1. Wash gravid adults and eggs from plates set up during “animal synchronization” step with 2–3 mL of M9 reused between 3 plates (e.g., wash plate 1 with M9, transfer M9+embryos to plate 2, repeat with plate 3) into a 15 mL tube. Ensure all animals and eggs are removed from the plates.
2. Centrifuge at 3,000 g for 1 min and remove supernatant containing bacteria (some bacteria will be pelleted, this is normal).
3. Wash with 1 mL of M9 centrifuging at 3,000 g for 1 min to remove excess bacteria (optional but recommended).
4. After the wash, aspirate down to the worm and egg pellet.
5. Add 1 mL of bleach solution (see “bleach solution” for preparation) and vortex for 2 min.

***Note:*** Animal carcasses will still be visible in addition to eggs.

6. Centrifuge at 3,000 g for 1 min, remove bleach solution taking care not to remove the pellet.
7. Add 1 mL of M9, vortex to mix and centrifuge at 3,000 g for 1 min (repeat 3 times).
8. After the final wash, aspirate down to about 100 μL volume.
9. Mix vigorously with a 1,000 μL pipette and distribute eggs into a corner of an OP50 seeded NGM plate.

***Note:*** Each tube has enough eggs for multiple 60 mm NGM+OP50 plates. Ensure plates are not overcrowded, roughly 500 eggs per 60 mm NGM+OP50 plate.

10. You will need 10 plates per condition for the exposure (e.g., if doing control, 0.05% ethanol, and 0.5% ethanol conditions, you will need 30 synchronized plates.)
11. These animals will reach the L4 stage in roughly 50–52 h at 20°C.

### Ethanol exposure

**Timing: 48 h**

This step describes a liquid culture ethanol exposure paradigm for snRNA-seq applications.

**Figure 2:**
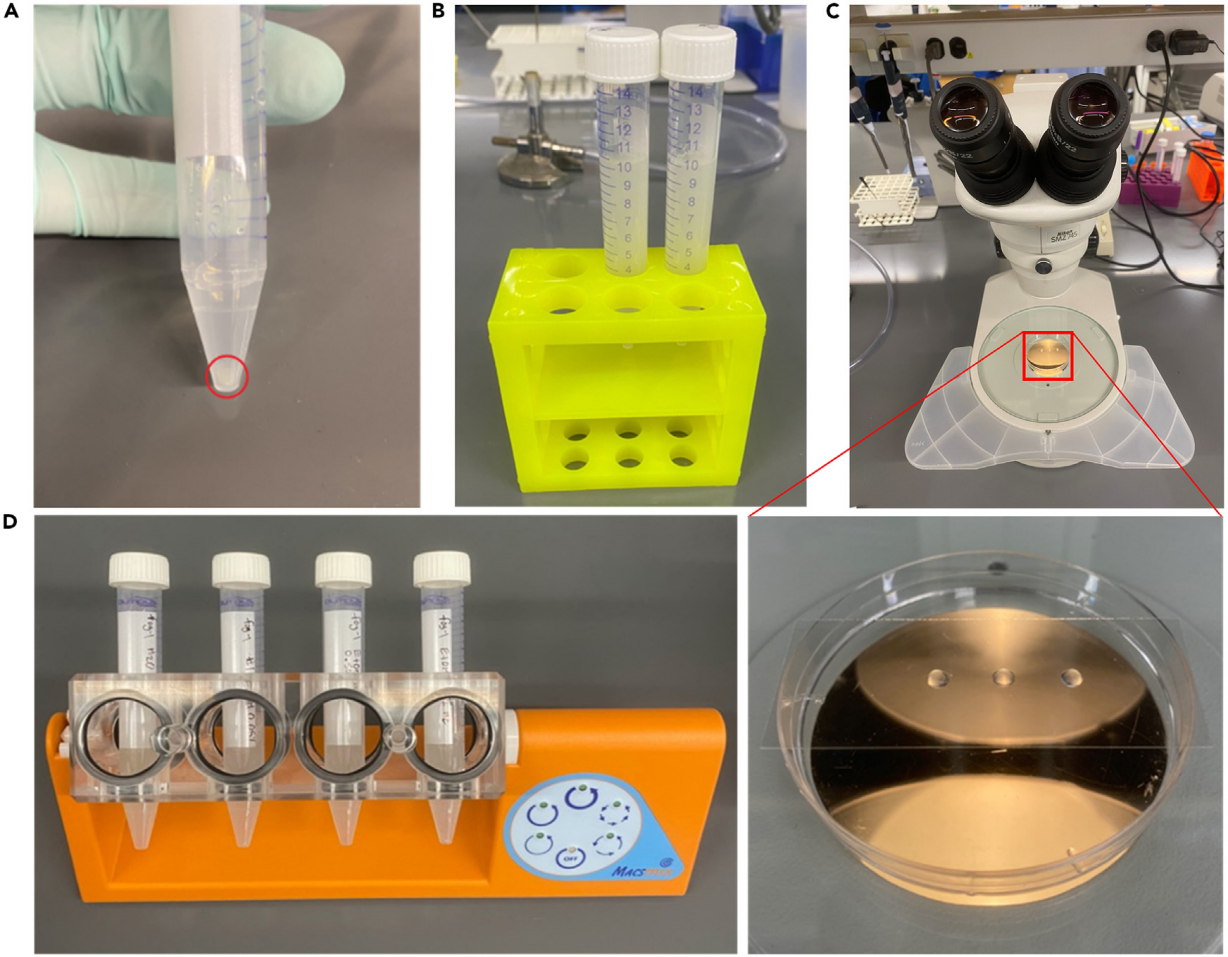
Liquid culture ethanol exposure setup Wash L4 animals off of plates using M9 and place in 15 mL conical tubes, allowing to settle by gravity (A). The pellet is then evenly distributed among two 15 mL conical tubes and filled up to 10 mL with M9 + 10 mg/mL OP50 (B). Three 5 μL samples from each well mixed tube are taken to attain a worm count/tube (C). Each exposure condition is then added to its own 15 mL tube and placed on a rotator (D).
18. To each tube, add 1 mL of 100 mg/mL OP50 + M9, and fill with M9 to a final volume of 10 mL (Figure 2B).

***Note:*** You should end up with a total of four 15 mL tubes containing animals in 10 mg/mL OP50 + M9.

12. Label three 15 mL tubes as “1”, “2” and “3”.
13. Place 4 mL of M9 into each of the three tubes.
14. Wash plates containing L4 animals with M9 using a 1,000 μL pipette (use roughly 2 mL of M9 per 3–4 plates), being careful not to remove dead carcasses from the previous bleaching step, and place into tube “1”.
15. Allow the animals to settle for around 5 min, then transfer the pellet (∼1 mL) into tube “2”.
16. Allow the animals to settle again, and transfer into tube “3”.
17. Allow animals to settle for around 5 min (Figure 2A), and divide each pellet evenly between two 15 mL tubes using a glass Pasteur pipette to prevent animals sticking (e.g., 2 pellets into four 15 mL tubes in total).

19. Quickly vortex each tube and take 5 μL of liquid placed onto a glass slide (average over 3 samples). Ensure that there is no more than an average of 4 worms per 5 μL sampled (Figure 2C).

***Note:*** If the density is too high, dilute by adding additional 10 mg/mL OP50 + M9 until in the proper range. If the density is too low, allow the animals to settle and remove liquid, ensuring you have enough volume to expose all conditions.

**CRITICAL:** Animals at too high a density will starve.

20. Prepare two 15 mL tubes per condition. In this case,
  a. 2 tubes for Control: 10 mL worms in 10 mg/mL OP50 + M9.
  b. 2 tubes 0.05% ethanol: 5 μL 100% ethanol + 10 mL worms in 10 mg/mL OP50 + M9.
  c. 2 tubes 0.50% ethanol exposure: 50 μL 100% ethanol + 9.95 mL worms in 10 mg/mL OP50 + M9.

**CRITICAL:** Make dilutions of ethanol fresh at room temperature, do not store. Ethanol 200 proof should be stored sealed at room temperature only up to a month after opening.

21. To each 15 mL tube, add up to 5 mL of previously prepared worms in 10 mg/mL OP50 + M9 leaving room to add ethanol at the proper concentration.

**CRITICAL:** Ensure worms have not settled, as this will create an uneven distribution.

22. Rotate slowly (∼10 rotations/min) at 20°C for 24 h (Figure 2D).

**Pause point:** Animals will incubate for 24 h.

23. After 24 h
  a. Near a Bunsen burner open the tubes to aerate for 5 min.
  b. Add 500 μL of 100 mg/mL OP50 + M9 into each tube to prevent starvation.
  c. Continue to rotate at 20°C for an additional 24 h (48 h total).

**Pause point:** Animals will incubate for 24 h.

### Gravid bleach of exposed P0 for the F1 generation

**Timing: 1 h**

**Figure 3:**
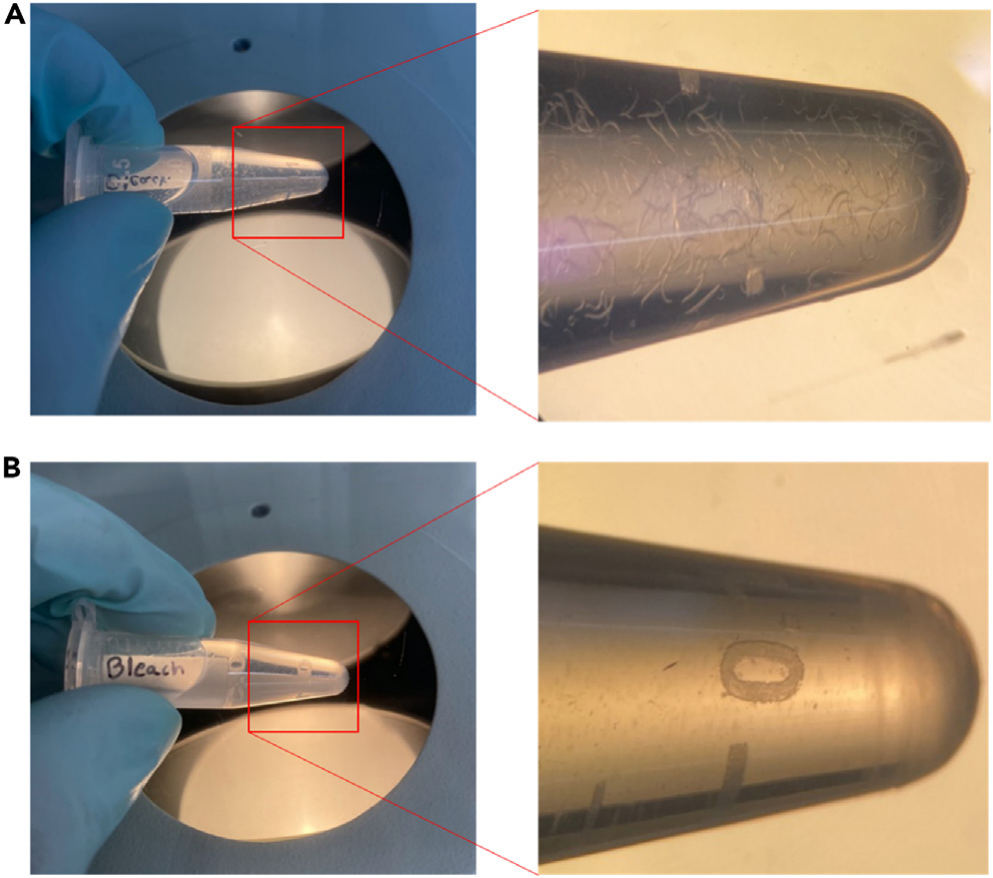
*C. elegans* gravid adult bleach for post-exposure synchronization Post exposure animals washed off plates should be at a high density with animals easily visible (A). After bleaching, ensure that no bodies are present to ensure the tightest synchronization. Embryos should still be visible under a dissecting microscope (B).

This step will synchronize animals in preparation for nuclear isolation (Figure 3).

24. After removing 15 mL tubes from the rotator, allow animals to settle for 5 min.
25. Transfer each pellet into a separate 1.5 mL tube (one tube per condition) with a low bind pipette or glass Pasteur pipette (Figure 3A).

***Note:*** Make sure to keep each condition separate.

26. Wash with 1 mL M9 to remove excess bacteria, spinning tubes at 3,000 g for 1 min.
27. After the wash, add 1 mL bleaching solution and vortex for 6–6.5 min. Eggs should be present, and bodies should be dissolved after beaching (Figure 3B). The longer duration of the bleaching step helps manage the larger animal input.

**CRITICAL:** After bleaching, ensure carcasses are not present. Be careful not to bleach for too long as this will kill the eggs that are recovered.

28. Quickly, wash 3 times with 1 mL M9. Spin at 3,000 g for 1 min after each wash.
29. After the final wash, aspirate each tube to ∼200 μL and distribute onto fresh NGM plates (about 10 μL/ NGM + OP50 60 mm plate, ∼ 500 eggs).
  a. Incubate at 20°C until animals reach the L4 stage (approximately 50–52 h).

***Note:*** Each tube has enough eggs for multiple OP50 NGM plates. Ensure plates are not overcrowded.

***Note:*** If culturing worms to the F3 generation, some F1 plates from each condition will need to be put aside to obtain the F2 generation, and similarly, some F2 plates will need to be set aside to obtain the F3 generation.

30. If you are collecting F1 nuclei from JK560 animals:
  a. After roughly 50–52 h post bleach, place half of the L4 plates per condition at 25°C for 24 h prior to nuclei dissociation to prevent sperm production.
  b. Otherwise, keep bleached animals at 20°C until they are Day 1 adults.

***Note:*** Save the remaining half of the plates at 20°C until ready to proceed to “**Step 31**”.

### Filtering out F1 adults to synchronize F2 L1 animals (repeat for F3 L1s)

**Timing: 1 h**

This step allows animals in the F2 generation to be synchronized without the need for a gravid adult bleach. This step can be repeated with the F2 adults and L1s to obtain a synchronized F3 population if necessary.

**Figure 4:**
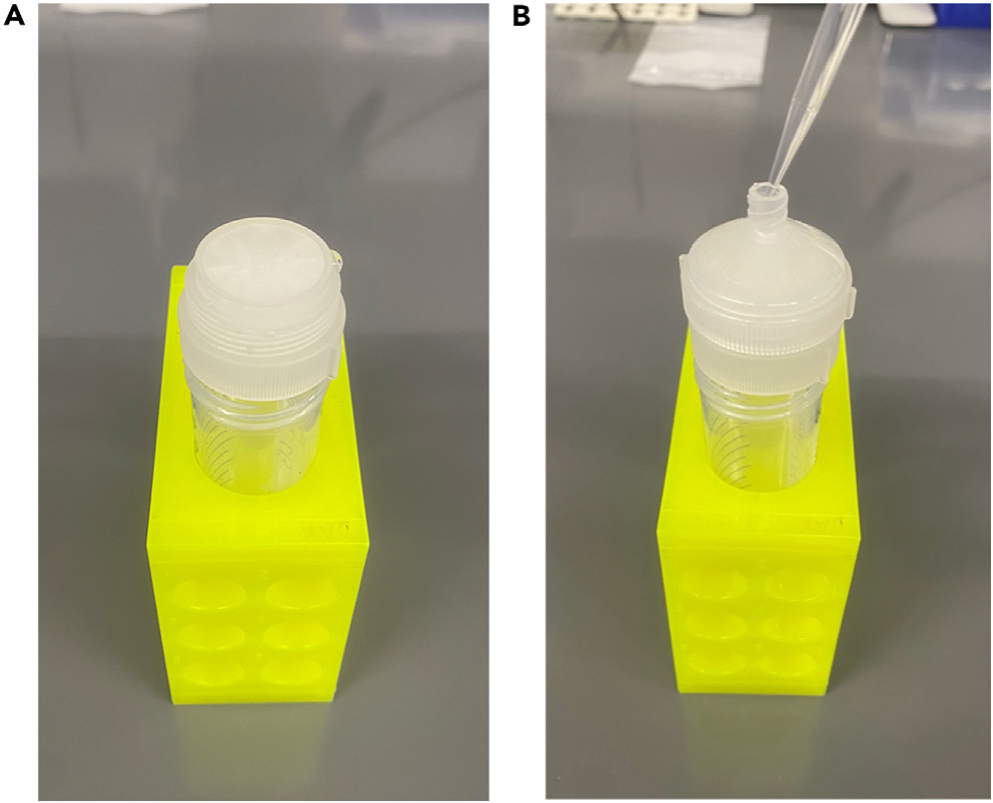
Filtering to isolate and synchronize L1 animals Prewet 40 μm filter to ensure proper position and function (A). Use a pipette to slowly filter animals washed off of plates with M9. Run through M9 will contain L1 animals in the 50 mL tube.
32. When the F2 animals are egg laying adults (∼96 h, 20°C), with L1s visible on the plates repeat **“Step 31”** to obtain a synchronized F3 generation.

***Note:*** Ensure all conditions remain separate and when plating L1s ensure plates are not overcrowded.

***Note:*** Pre-wetting the filter with M9 will ensure the filter stays in the filter holder properly.

31. Filter 7-10 60 mm plates per condition using a 10 μm filter in a sterile Swinnex filter holder, using M9 to wash the animals through into a 50 mL tube (Figures 4A and 4B).
  a. Wash animals from NGM plates with M9.
    ***Note:*** Visually inspect each plate to ensure L1s are present before washing off the animals.
  b. Run through 10 μm filter and follow with 3 times 1 mL M9 rinses into a 50 mL tube.
  c. Only L1s will be washed through the filter.
  d. Allow L1s to settle (about 5 min).
  e. Place animals onto fresh OP50 NGM 60 mm plates, taking care not to overcrowd the plates (∼500 animals/plate, 10 plates per condition).

### Nuclei dissociation

**Timing: 2 h**

After animals have been synchronized at the adult stage, the following steps will allow dissociation of individual nuclei from whole worms for downstream 10× Genomics sequencing.

**Figure 5:**
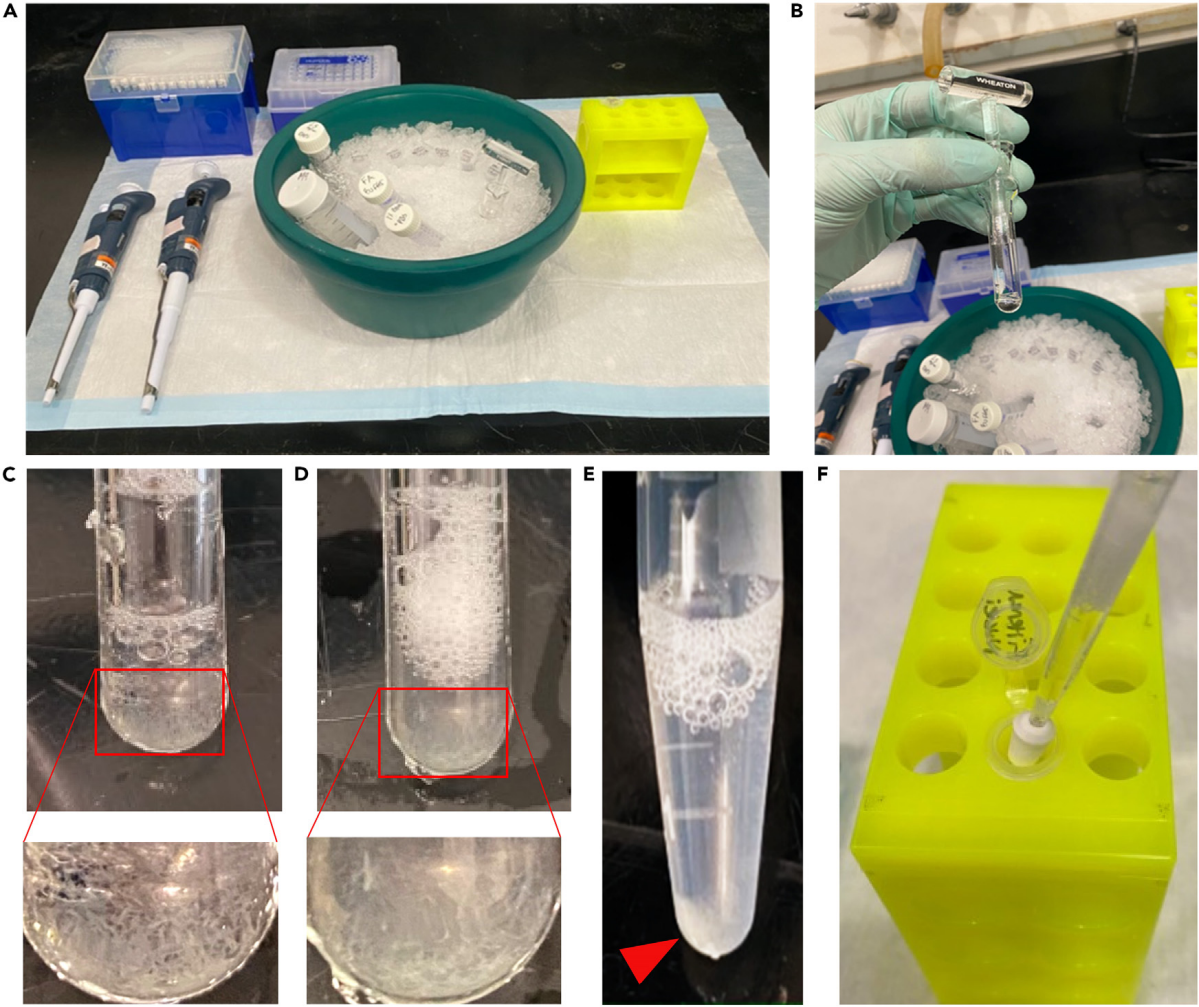
Nuclear extraction setup Ensure that sufficient ice is available to keep all reagents cold. Ensure the environment is sterile and RNase free in a 4°C environment (A). Ensure that Modified FA buffer is not able to overflow from the Dounce tissue grinder and keep contents cold to preserve nuclei integrity (B). Add the worm pellet to the Dounce homogenizer (C) and homogenize the samples (D). Centrifuge the homogenized samples (E), the pellet (arrow) contains cellular debris. Finally, filter the supernatant through a Flowmi filter (F).
34. Wash worms from plates with M9 (roughly 2 mL per 3–4 plates) into 15 mL tubes.
  a. Try to limit bacteria transfer while washing the plates.
  b. If too much bacteria transfers, allow the worms to settle (∼5 min).
  c. Transfer the pellet to a new tube and with 5 mL fresh M9.
  d. Repeat “**Steps 34b-c**” 3 times.

***Note:*** Bacteria will negatively affect sequencing results; ensure bacteria are fully removed from the animals before continuing.

33. Ensure all reagents are prepared ahead of time (Figure 5A).
  **CRITICAL:** Perform all centrifugation steps at 4°C. Perform all steps in RNase free conditions. Keep all samples and reagents on ice. Minimize pipetting and handling time to avoid nuclei lysis or clumping (Figures 5A–5F).
  a. 1.2 mL per condition Modified FA Buffer (store on ice, use the same day).
  b. 2.0 mL per condition of 1× PBS with 1% BSA and RNase inhibitor (store on ice, use the same day).
  c. Clean, label with each condition, and pre-chill homogenizers at -20°C.
  d. Ensure all tubes are labeled and the 10× Genomics sequencing facility is ready to receive samples. For each condition, the labels should be:
    i. Homogenized sample.
    ii. Pooled nuclei.
    iii. New nuclei.
    iv. Filtered nuclei.
    v. 10× Genomics submission (marked with each condition).
    vi. 96 well plate for BD Celesta cell analyzer equipped with a 405 nm Laser.
  e. Sterilize the work area and treat with RNase Zap.
    ***Note:*** Each exposure condition should get its own set of tubes; never mix nuclei from different exposure conditions!

35. Allow the worms to settle into a pellet (∼5 min), and transfer to a low bind 1.5 mL tube using a glass Pasteur pipette.
36. Centrifuge at 1,300 g for 1 min, remove M9 and wash with 1 mL M9 (Repeat for 3 total washes).
  a. Be careful not to disturb the worm pellet.
37. Starve animals for 30 min on a rotator in 1 mL fresh M9.
  a. This step will evacuate the gut of bacteria improving sample quality.
38. Allow the worms to settle for 10 min to obtain a 30 μL pellet.
39. Place 450 μL Modified FA buffer into a Wheaton Dounce homogenizer (Figure 5B).
40. Transfer a 30 μL worm pellet into a Dounce homogenizer containing 450 μL of Modified FA buffer (Figure 5C).
41. Homogenize each sample with an up and down corkscrew motion (10 strokes).
  a. Be careful not to spill sample and try to limit the creation of excess bubbles (Figure 5D).
42. Transfer Modified FA buffer with nuclei from the Dounce homogenizer to a fresh low bind 1.5 mL tube and centrifuge at 100 g for 1 min at 4°C (Figure 5E).
  a. The supernatant will contain nuclei.
  b. The pellet will contain debris and waste.
43. Remove the supernatant and place it into 1.5 mL tube labeled “Pooled Nuclei”.
44. Resuspend the pellet with 350 μL Modified FA buffer in the same 1.5 mL tube.
45. Homogenize the resuspended pellet with a sterile, RNase free pestle (plastic) in the 1.5 mL tube, using 10 corkscrew motions.
46. Centrifuge at 100 g for 1 min to pellet debris.
47. Remove the supernatant and place it into “Pooled Nuclei” tube.
48. Resuspend the pellet again in 350 μL Modified FA buffer in the same 1.5 mL tube.
49. Homogenize with a plastic pestle with 10 corkscrew motions.
50. Centrifuge at 100 g for 1 min to pellet any remaining debris.
51. Transfer the supernatant into “Pooled Nuclei” 1.5 mL tube.
52. Pellet the nuclei by centrifuging at 500*g* for 4 min.

***Note:*** Ensure that different conditions remain separate.

***Note:*** After this step nuclei will be in the pellet.

53. Remove the supernatant (modified FA buffer), making sure to leave the nuclei pellet behind.

***Note:*** If the pellet isn’t visible, aspirate supernatant down to 0.1 mL.

54. Resuspend the nuclei pellet using 1 mL of 1% BSA in 1× PBS, pH 7.4 with RNase inhibitor.
55. Centrifuge the nuclei at 500*g* for 1 min.
56. Remove supernatant and resuspend the pellet to a total volume of ∼900 μL with 1% BSA in 1× PBS pH 7.4 with RNase inhibitor.

***Note:*** Resuspend in less volume if the pellet is barely visible (∼700 μL total volume).

57. Filter with a Flowmi 40 μm filter to remove extra debris and clumped nuclei (Figure 5F).
  a. Transfer to a final tube for 10× Genomics sequencing.
58. Use a BD Celesta cell analyzer with 96 well plate and 405 nm laser compatibility or similar cell analyzer for the final nuclei counts.
  a. From each “Final Nuclei” tube, remove 450 μL of filtered nuclei for DAPI staining. Make sure that the nuclei are well mixed to ensure you are not over or under sampling the nuclei count.
    **CRITICAL:** The remaining nuclei samples will be sent in for 10× Genomics snRNA-sequencing. Ensure they are kept on ice to prevent damage or degradation.
  b. Stain with DAPI by adding 2 μL of 2.5 mg/mL DAPI (final concentration 11 μg/mL).
    ***Note:*** While flow cytometry tends to over-estimate nuclei, this is our recommended methodology for consistent counts. Additionally, we have found cell counters to be inconsistent with *C. elegans* nuclei due to the heterogeneous nature of their size and shape.
  c. Prepare a 96 well plate, with each condition in technical triplicates.
    i. Load 150 μL of sample per well.
      ***Note:*** An accurate count is essential for proper submission to 10× Genomics and accurate sequencing results.
  d. Use the total count number for your 10× Genomics submission.
    ***Note:*** The remaining nuclei without DAPI added will be submitted for 10× Genomics sequencing.
    ***Note:*** Ensure you have an accurate estimate of your number of nuclei to get the cleanest sequencing results.
    ***Note:*** You can use a fluorescent microscope to check for clumping by mounting DAPI stained nuclei onto Vectashield or similar mount (∼3–5 μL of nuclei suspension) (Figure 6).
    **Figure 6:**
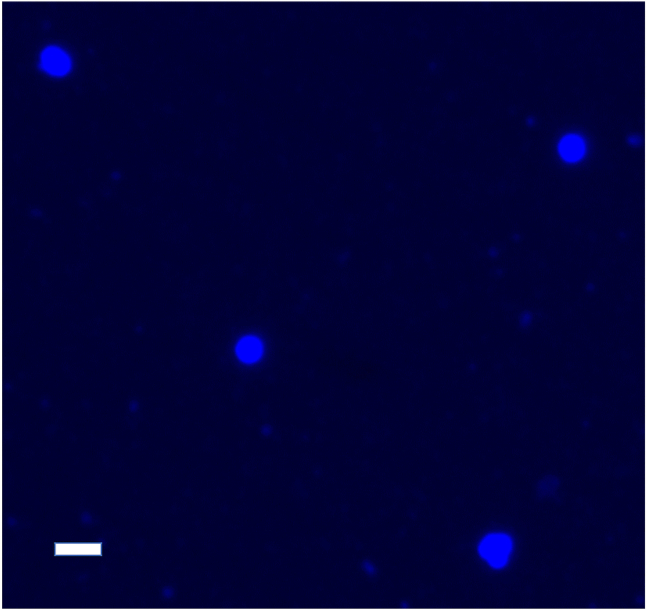
Ensure nuclei are not clumped and intact with DAPI staining Scale bar = 10 μm.
  e. If nuclei samples are clumping, run the samples through an additional Flowmi 40 μm filter.
59. Deliver the samples for library preparation and sequencing as soon as possible to ensure the sample quality doesn’t degrade (here we used the 10× Genomics platform).

### Data processing

**Timing: variable**

This step will briefly outline the bioinformatic pipeline utilized to process raw 10× Genomics snRNA-seq data to remove low quality datapoints stemming from debris containing droplets and ambient RNA contamination (detailed version is available in Truong et al.*, 2023*^1^).

60. Resources to process raw snRNA-seq data.
  a. First, snRNA-seq raw reads were:
    i. Demultiplexed and aligned to the *C. elegans* ENSEMBL ce10 transcriptome utilizing Cell Ranger, using “include-introns” flag to align nucleus reads.
    ii. Sample quality control was determined through checking Cell Ranger reports such as: saturation, mapping rate and sample clustering.
  b. To remove debris contamination and RNA contamination we utilized:
    i. DIEM to remove droplets with extranuclear contamination.^11^
    ii. SOUPx to identify ambient RNA and correct the expression reads for remaining droplets.^12^
    iii. Filter out low-quality droplets (droplets with identified genes less than 300 or more than 3,500, UMIs more than 15,000 or mitochondrial reads more than 20%). Threshold should be determined based on visualization of QC plots.
  c. Canonical correction analysis (CCA) in Seurat v3^13^ was utilized to remove batch effect across samples.
  d. Principal component analysis (PCA) and Louvain clustering^14^ were further used to identify clusters.
  e. We recommend visualizing cell clusters with either t-distributed stochastic neighbor embedding (t-SNE) plot or Uniform Manifold Approximation and Projection (UMAP). The former works better to identify different subpopulation within cluster and latter works better to identify distinct clusters.
  f. We utilized known biomarkers (e.g., *tra-2* for the germline, *unc-25* for GABA neurons-specific markers; see Truong et al., 2023^1^ Figure 2 and Supplementary Data S2) and used comparison and dot-heatmap visualization to determine cluster identity.
  g. Differential expressed genes (DEGs) were identified based on Monocle^15^ pipeline with batch effect variable involved and corrected with FDR <5%.
  h. Differential expressed genes of different clusters were further compared with different ontological gene sets through enrichment analysis (FDR <5%).
    i. Tissue enrichment.
    ii. Gene Ontology.
    iii. Wormbase phenotype.^16^

Cross-reference the previous outputs with *in situ* expression for top transcripts utilizing the Nematode Expression Pattern Database (NEXTDB https://nematode.nig.ac.jp).

61. Validate differentially expressed genes (DEGs) through a secondary analysis method, we recommend single molecule fluorescence *in situ* hybridization (smFISH).^1^^,^^17^

## Expected outcomes

The technique should generate approximately 1,200 nuclei per 4,000 worm sample. Following clustering, cell types should resolve into clusters as shown here:

https://singlecell.broadinstitute.org/single_cell/study/SCP922/single-nucleus-resolution-mapping-of-the-adult-c-elegans-and-its-application-to-elucidate-inter-and-trans-generational-response-to-alcohol#study-visualize.

## Limitations

This protocol was solely tested in adult hermaphrodite *C. elegans.* It is probable that the number of input animals required to examine younger stages (i.e., larval stages) would need to be increased from our original protocol. Additionally, the mechanical disruption to the worms can potentially lead to under sampling of certain cell types; however, we were able to observe clusters representing all tissue types.

## Troubleshooting

### Problem 1

Issue during maintenance of the animals during scaling up of cultures: the animals have starved and there is no more OP50 when in liquid culture or on plates.

(Related to sections “animal synchronization”, rapid bleach to synchronize animals for exposure”, “ethanol exposure” and “gravid bleach of exposed P0 for the F1 generation”).

### Potential solution

• Add less animals to each plate or into the liquid culture.
• Estimate the number of animals you are using to ensure consistency (see step 19).

### Problem 2

Animal carcasses are still present after gravid adult bleach.

(Related to section “gravid bleach of exposed P0 for the F1 generation”).

### Potential solution

• Double the amount of bleaching solution. If too many animals are in the tube, it can affect the quality of the bleaching output.
• Reduce the number of animals being bleached.
• Extend the bleach time by 30 s; however, bleaching for too long may kill the embryos.

### Problem 3

Unable to grow enough animals to perform nuclear isolation.

(Related to section “animal aynchronization”).

### Potential solution

• Strains with lower fecundity due to their genetic background may require either:
  ○ More plates OR larger plates with extra OP50 to increase population size.
  ○ Place more animals per plate initially if the plates do not starve when more animals are used. (e.g., instead of 5–6 L4s/ 6 cm plate attempt to put 10–12 L4s/ 6 cm plate).

### Problem 4

Isolated nuclei form clumps making a poor input for single nucleus sequencing (related to section “nuclei dissociation”).

### Potential solution

• The best way to limit nuclei clumping is to proceed as quickly as possible through the “nuclei dissociation” section. We found that extra time during this step promotes both the likelihood of clumping and the deterioration of the nuclei quality.
• Make sure that all reagents and material are prepared in advance in addition to ensuring that samples can be rapidly delivered for sequencing.
• Use computational strategies such as those described above and in Truong et al.*, 2023*^1^ to remove doublets post-sequencing.

### Problem 5

Nuclei count too low for submission to 10× Genomics.

(Related to section “nuclei dissociation”).

### Potential solution

• Resuspend the final nuclei pellet in a smaller volume.
• Increase the animal input used in “nuclei dissociation”.
• Ensure the supernatant is being collected in steps 43, 47 and 51.
• In step 52, the nuclei are in the pellet.

## Resource availability

### Lead contact

Further information and requests for resources and reagents should be directed to and will be fulfilled by the lead contact, Patrick Allard (pallard@ucla.edu).

### Materials availability

This protocol did not generate new unique reagents.

### Data and code availability

All raw data is accessible on NCBI’s Gene Expression Omnibus (GEO), at: https://www.ncbi.nlm.nih.gov/geo/query/acc.cgi?acc=GSE208229.

The data is also available through the Broad Single Cell Portal: https://singlecell.broadinstitute.org/single_cell/study/SCP922/single-nucleus-resolution-mapping-of-the-adult-c-elegans-and-its-application-to-elucidate-inter-and-trans-generational-response-to-alcohol.
